# Miscibility Tuning for Optimizing Phase Separation and Vertical Distribution toward Highly Efficient Organic Solar Cells

**DOI:** 10.1002/advs.201900565

**Published:** 2019-05-22

**Authors:** Lifu Zhang, Nan Yi, Weihua Zhou, Zoukangning Yu, Feng Liu, Yiwang Chen

**Affiliations:** ^1^ College of Chemistry Nanchang University 999 Xuefu Avenue Nanchang 330031 China; ^2^ Institute of Polymers and Energy Chemistry (IPEC) Nanchang University 999 Xuefu Avenue Nanchang 330031 China; ^3^ School of Material Science and Engineering Nanchang University 999 Xuefu Avenue Nanchang 330031 China; ^4^ School of Chemistry and Chemical Engineering Shanghai Jiaotong University 800 Dongchuan Shanghai 200240 China

**Keywords:** crystallization, interaction parameter, morphology, ternary devices, vertical phase separation

## Abstract

Blending multidonor or multiacceptor organic materials as ternary devices has been recognized as an efficient and potential method to improve the power conversion efficiency of bulk heterojunction devices or single‐junction components in tandem design. In this work, a highly crystalline molecule, DRCN5T, is involved into a PTB7‐Th:PC_70_BM system to fabricate large‐area organic solar cells (OSCs) whose blend film thickness is up to 270 nm, achieving an impressive performance of 11.1%. The significant improvement of OSCs after adding DRCN5T is due to the formation of an interconnected fibrous network with decreased π–π stacking and enhanced domain purity, in addition to the optimized vertical distribution of PTB7‐Th and PC_70_BM, producing more effective charge separation, transport, and collection. The optimized morphology and performance are actually determined by the miscibility in different components, which can be quantitatively described by the Flory–Huggins interaction parameter of −0.80 and 2.94 in DRCN5T:PTB7‐Th and DRCN5T:PC_70_BM blends, respectively. The findings in this work can potentially guide the selection of an appropriate third additive for high‐performance OSCs for the sake of large‐area printing and roll‐to‐roll fabrication from the view of miscibility.

## 1 Introduction

Organic solar cells (OSCs) play an important role over the past two decades, which have grown rapidly with interpenetrating network structure of active layer, and this structure is also known as bulk heterojunction (BHJ).^1^, ^2^, ^3^ It has been recognized as a great promising substitute for silicon‐based photovoltaic with advantages of light weight, inexpensive cost, and large‐area flexible preparation with roll‐to‐roll (R2R) process. Besides understanding the internal mechanism of binary OSCs deeply, more and more efforts were paid to improve the performance of OSCs via tuning absorption and energy level of novel donor^4^, ^5^, ^6^, ^7^, ^8^, ^9^, ^10^, ^11^, ^12^ and acceptor^13^, ^14^, ^15^ materials, optimizing processing methods,^16^, ^17^, ^18^, ^19^ interfacial engineering,^20^ and innovative device architectures.^14^, ^21^, ^22^, ^23^, ^24^, ^25^, ^26^, ^27^

As an efficient and new type of device structure, ternary OSCs generally consist of multidonor and one acceptor or one donor and multiacceptor. The third component is usually combined with the host single‐junction system to provide complementary absorption, tune recombination behavior, and optimize the morphology of active layer.^28^, ^29^, ^30^ Up to now, the best performance of ternary OSCs has been reported to be over 14%.^13^ What is more, another novel device, tandem OSC,^24^ consisting of two or more sub‐cells has achieved a record‐breaking efficiency of 17.3%.^25^ Although both ternary OSCs and tandem OSCs could improve the power conversion efficiency (PCE) value to about 15%, the ternary strategy possessing simplicity of processing active layer exhibits great potential in R2R technology.^31^ And the ternary OSCs literatures have been widely published in recent years including fullerene and non‐fullerene systems.^6^, ^32^, ^33^, ^34^ Unfortunately, most of the research work about how to select appropriate third additive is based on the trial and error strategy, which is a process of labor and time consumption. The light absorption, energy level alignment, energy transfer, crystallization as well as miscibility should be taken into consideration during the fabrication of ternary OSCs.^29^ Especially, many researchers have realized the importance of miscibility in ternary OSCs,^28^, ^35^, ^36^, ^37^ such as the appearance of Förster resonance energy transfer,^38^ distribution of third additive on the interfaces of donor and acceptor,^39^ formation of alloy with donor or acceptor,^36^, ^37^, ^40^ etc. However, the effect of miscibility on the fabrication of thick blend films ternary OSCs, in addition to the vertical distribution of donor and acceptor remains to be explored.^31^, ^41^ The relative research work about how to quantitatively describe the miscibility in ternary systems has scarcely been reported.

For example, it is crucial to increase the active layer thickness of ternary OSCs without damaging the parameters of devices, which is contributive to the fabrication of large area OSCs via printing techniques. Nevertheless, the majority of ternary components show their highest performance at the appropriate blend films thickness (about 100 nm), further increasing the film thickness eventually would bring about the sharp reduction in PCE values.^42^ There are multiple reasons for the poor performance of ternary OSCs with thicker active layer. When the crystallization of donor or acceptor is disturbed by the incorporation of third additive,^6^, ^43^ the decreased domain purity may lead to the depression of charge mobility and the enhanced charge recombination, causing the severe reduction of PCE and fill factor (FF).^38^ Furthermore, as the blend film thickness increased, the effect of surface energy of substrates on the vertical distribution of donor and acceptor lessened, leading to the unfavorable enrichment of donors or acceptors at cathode and anode, which is harmful to the charge collection in the respective electrodes.^21^, ^44^ Weather the substrate character is hydrophobic or not, it is still unknown that what determines the morphological stability of thick active layer in the vertical direction.

With the efforts of researchers, it has already been proved that the introduction of crystalline small molecules such as BTR (benzodithiophene terthiophene rhodanine) donor involved into the PTB7‐Th:PC_70_BM binary system could achieve the significant improvement in PCE, as well as the fabrication of thick active layer films approaching to 250 nm. As stated, the enhancement is due to the decreased π–π stacking distance with higher domain purity and charge transport. Although the authors mentioned the good miscibility between PTB7‐Th and BTR, the impact of miscibility on the corresponding performance of the solar cells was not discussed.^31^ Zhang et al. found that the *p*‐DTS(FBTTH_2_)_2_ could also effectively increase the PCE value of PTB7‐Th:PC_70_BM, stating the formation of miscible alloy between third additive and polymer by the differential scanning calorimetry (DSC) and wide‐angle grazing‐incidence X‐ray scattering (GIWAXS) analysis.^36^, ^45^ Although the miscibility is recognized to be an important factor in determining the performance of cells, no detailed quantitative description of miscibility is presented.^28^, ^35^ Thanks to the Ade's deep investigation, the Flory–Huggins interaction parameter χ was calculated by the melting‐point depression method,^46^ demonstrating that the two polymers of FTAZ and PDPP3T were completely miscible.^22^ Unfortunately, the PCE value of ternary system is much lower than the binary system. It should point out that the χ value lower than 0.5 demonstrates that the two components are totally miscible, whereas the χ value above 1.5 reveals that they are immiscible.^47^, ^48^ The use of Flory–Huggins interaction parameter could quantitatively describe the degree of miscibility between two components.^45^, ^49^ The χ value could be calculated via the solubility parameter or determination of phase diagram by the use of small‐angle neutron scattering, secondary‐ion mass spectrometry (SIMS), synchrotron radiation‐based scanning transmission X‐ray microscopy (STXM), and so on.^47^ It is noted that the solubility parameter method failed to describe the true miscibility between two components irrespective of the specific π–π interaction in conjugated polymer systems. The advanced SIMS and STXM techniques require the platform of advanced national laboratory, which is more complex than the DSC‐based technique.^49^ Therefore, the melting point depression method is more applicable in the field of miscibility determination. In our previous study, comparing with P3HT and P3OT, the P3BT with shorter side alkyl chain exhibited smaller χ values when combined with *p*‐DTS(FBTTH_2_)_2_ or PC_70_BM, and the small χ value enhanced PCE.^50^ The Flory–Huggins interaction parameter between third additive and donor or acceptor should all be taken into consideration to predict the effect of miscibility on the morphological evolution in the horizontal and vertical direction of the blend film.

In this article, the widely studied PTB7‐Th and PC_70_BM are chosen as the host system due to the high reproducibility and performance.^32^ The small molecule DRCN5T was selected as the extra additive attributing to the well‐matched energy level alignment and high crystallinity. It is founded that the involvement of DRCN5T in PTB7‐Th:PC_70_BM system could enhance the absorption, which is helpful for the preparation of thick active layer of 270 nm whose maximal PCE value is of 11.1%, comparing with the binary system of 7.9% (250 nm) and 8.8% (100 nm). Surprisingly, the larger area ternary devices with efficient area up to 18 mm^2^ could still retain PCE above 10% in contrast to the traditional devices with efficient area of 4 mm^2^. The excellent PCE of the ternary active layer system is demonstrated to be related with the miscibility of different components. The Flory–Huggins interaction parameter measured by DSC is calculated to be −0.80 and 2.94 in DRCN5T:PTB7‐Th and DRCN5T:PC_70_BM blends, respectively. The good miscibility between DRCN5T and PTB7‐Th facilitates the crystallization of PTB7‐Th to form the interconnected fibrous network, showing more face‐on crystals with higher domain purity. The aggregation of PC_70_BM molecules separates from PTB7‐Th phase because of the bad miscibility between DRCN5T and PC_70_BM, while more PTB7‐Th polymers with lower surface energy could distribute on the blend film superficially. The optimized distribution of PTB7‐Th and PC_70_BM in vertical and horizontal direction is beneficial to the large‐area, thick, and high performance OSCs. The investigation of the miscibility between different components could provide knowledge for modulating the active layer morphology effectively and precisely so as to achieve higher efficiency of OSCs.

## 2 Results and Discussions

**Figure 1:**
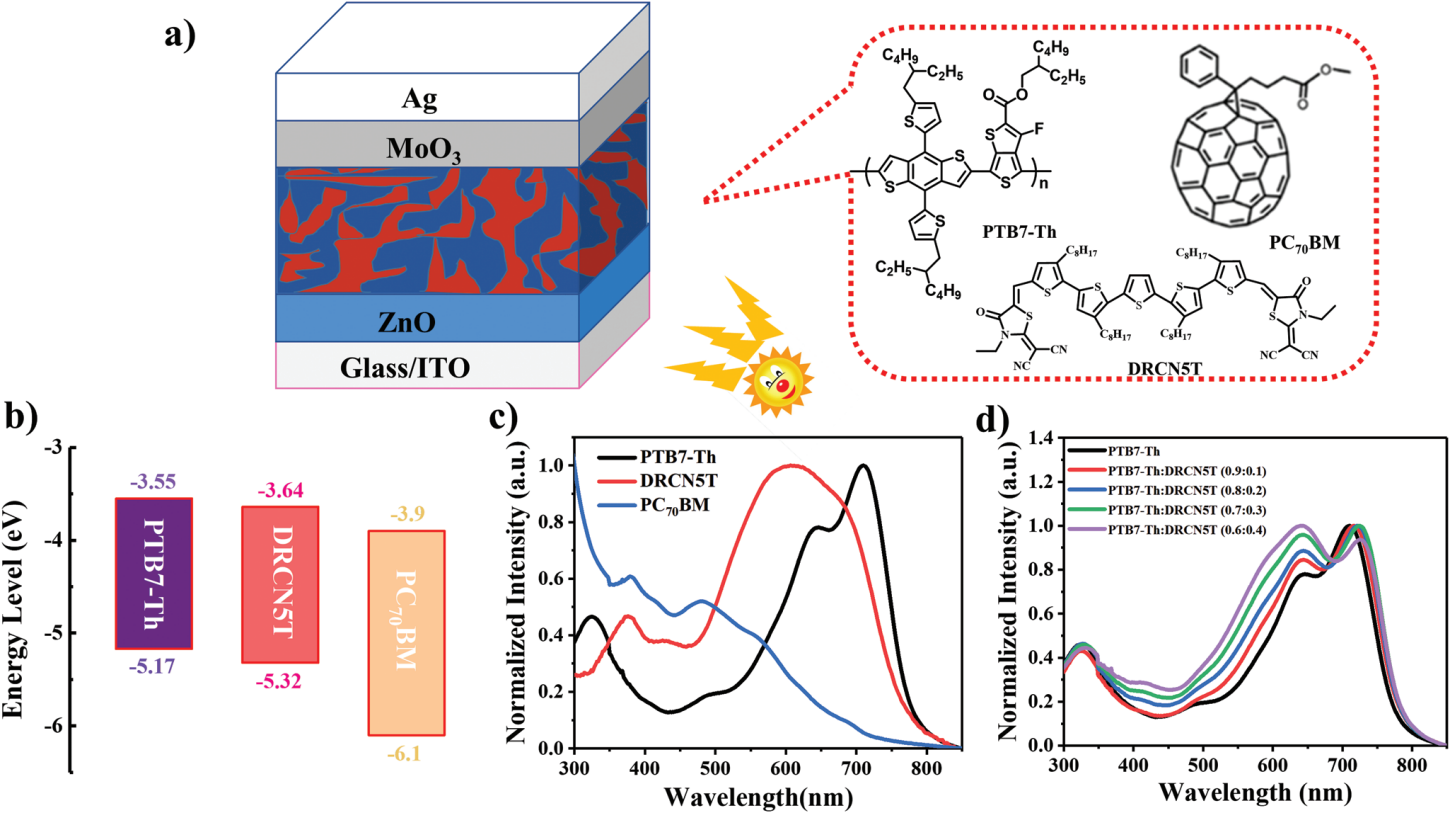
a) Schematic diagram of the inverted structure device. b) Energy levels diagram of BHJ materials. c) Normalized absorption spectra of neat PTB7‐Th, DRCN5T, and PC_70_BM films. d) Normalized absorption spectra of PTB7‐Th containing different amounts of DRCN5T.

The chemical structure, energy levels as well as the absorption spectrums of pristine PTB7‐Th, DRCN5T, and PC_70_BM are displayed in **Figure** **1**a–c. The highest occupied molecular orbital (HOMO) level and the lowest unoccupied molecular orbital level of DRCN5T are −5.32 and −3.64 eV, respectively,^51^ which forms an energy cascade with PTB7‐Th and PC_70_BM benefiting for charge separation and collection.^29^ It is observed that DRCN5T displays complementary absorption with PTB7‐Th and PC_70_BM from 450 to 700 nm. Hoping to investigate the impact of DRCN5T on the absorption behaviors, the binary PTB7‐Th:DRCN5T and ternary PTB7‐Th:DRCN5T:PC_70_BM blend films with different DRCN5T ratio are analyzed, as shown in Figure 1d and Figure S1 in the Supporting Information. With the increment of DRCN5T ratio in PTB7‐Th, the intensity of absorption increased gradually in the wavelength of 350–650 nm. In the PTB7‐Th:DRCN5T blend film (Figure 1d), the incorporation of DRCN5T leads to an obvious absorption peak shifting from 709.9 to 724.6 nm, demonstrating a significantly increased molecular ordering of PTB7‐Th, due to the specific interaction between them. Therefore, it is speculated that the crystallization of PTB7‐Th could be enhanced by the incorporation of DRCN5T.^31^ In addition, the enhancement of absorption is eventually beneficial to the increase of current density.^14^

**Figure 2:**
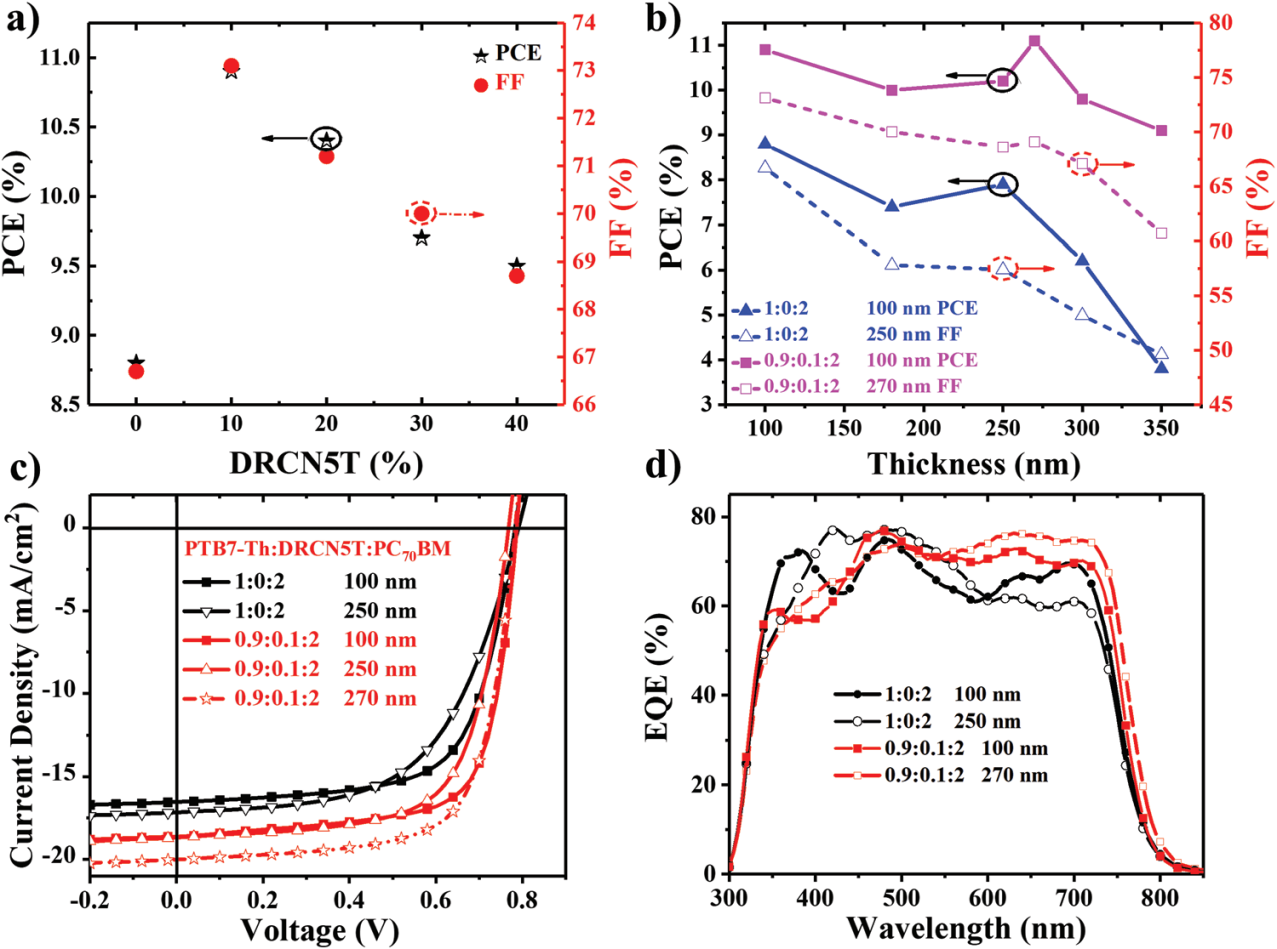
a) PCE and FF values of ternary OSCs with thin active layers versus different ratios of DRCN5T. b) PCE and FF of binary and ternary OSCs versus active layer thickness, c) *J*–*V* and d) EQE curves of PTB7‐Th:PC_70_BM binary and PTB7‐Th:DRCN5T:PC_70_BM ternary devices with active layers of different thickness.

The photovoltaic performance of both binary and ternary devices fabricating in the inverted structure of indium tin oxide (ITO)/ZnO/BHJ films/MoO_3_/Ag is shown in Figure S2a in the Supporting Information. For the purpose of systematically investigating the impact of DRCN5T ratio on the performance of devices, the weight ratio of donor to PC_70_BM is first kept constant at 1:2, and the thickness of the active layer is maintained at about 100 nm. As observed from **Figure** **2** and **Table** **1**, the PTB7‐Th:PC_70_BM presented a PCE value of 8.8%, a *V*_oc_ of 0.782 V, *J*_sc_ of 16.54 mA cm^−2^, and FF of 67.2%, respectively. After the incorporation of 0, 10, 20, 30, and 40 wt% DRCN5T into the binary PTB7‐Th:PC_70_BM system, the *J*_sc_ value first increased from 16.53 to 18.63 mA cm^−2^, and then reduced to 17.84, 17.13, and 16.99 mA cm^−2^, respectively. A similar trend of FF change versus DRCN5T ratio was also observed. It was noteworthy that the *V*_oc_ values of devices increased slightly with the increase of DRCN5T content, which was probably on account of the lower HOMO level of DRCN5T than that of PTB7‐Th.^52^ The ternary blending device with 10 wt% DRCN5T showed the highest PCE value of 10.9% with an FF of 73.2%, *V*_oc_ of 0.782 V, and *J*_sc_ of 18.88 mA cm^−2^, which is a remarkable achievement in the PTB7‐Th:PC_70_BM ternary systems.^53^

**Table 1:** Photovoltaic parameters of ternary OSCs with different DRCN5T contents

PTB7Th:DRCN5T:PC_70_BM	Thickness [nm]	*V*_oc_ [V]	*J*_sc_ [mA cm^−2^]	*J*_calcd_ [mA cm^−2^]^a)^	FF [%]	PCE_ave_ [%]^b)^	PCE_max_ [%]
1:0:2	100	0.782 ± 0.02	16.53 ± 0.11	16.2	66.7 ± 0.5	8.6 ± 0.2	8.8
1:0:2	250	0.78 ± 0.002	17.16 ± 0.04	16.4	57.4 ± 0.3	7.8 ± 0.2	7.9
0.9:0.1:2	100	0.787 ± 0.01	18.63 ± 0.25	18.3	73.1 ± 0.5	10.7 ± 0.2	10.9
0.9:0.1:2	250	0.78 ± 0.002	18.86 ± 0.07	17.95	68.6 ± 0.5	10.1 ± 0.1	10.2
0.9:0.1:2	270	0.78 ± 0.002	20.10 ± 0.07	19.05	69.8 ± 0.2	10.7 ± 0.4	11.1
0.8:0.2:2	100	0.788 ± 0.01	17.84 ± 0.32	17.2	71.2 ± 0.3	10.2 ± 0.2	10.4
0.7:0.3:2	100	0.790 ± 0.02	17.13 ± 0.46	16.7	70.0 ± 0.1	9.6 ± 0.1	9.7
0.6:0.4:2	100	0.798 ± 0.02	16.10 ± 0.20	16.4	68.7 ± 0.1	9.3 ± 0.2	9.5

^a)^ *J*_sc_ integrated from the EQE spectrum

^b)^ All average PCE values are obtained from ten devices.

**Figure 3:**
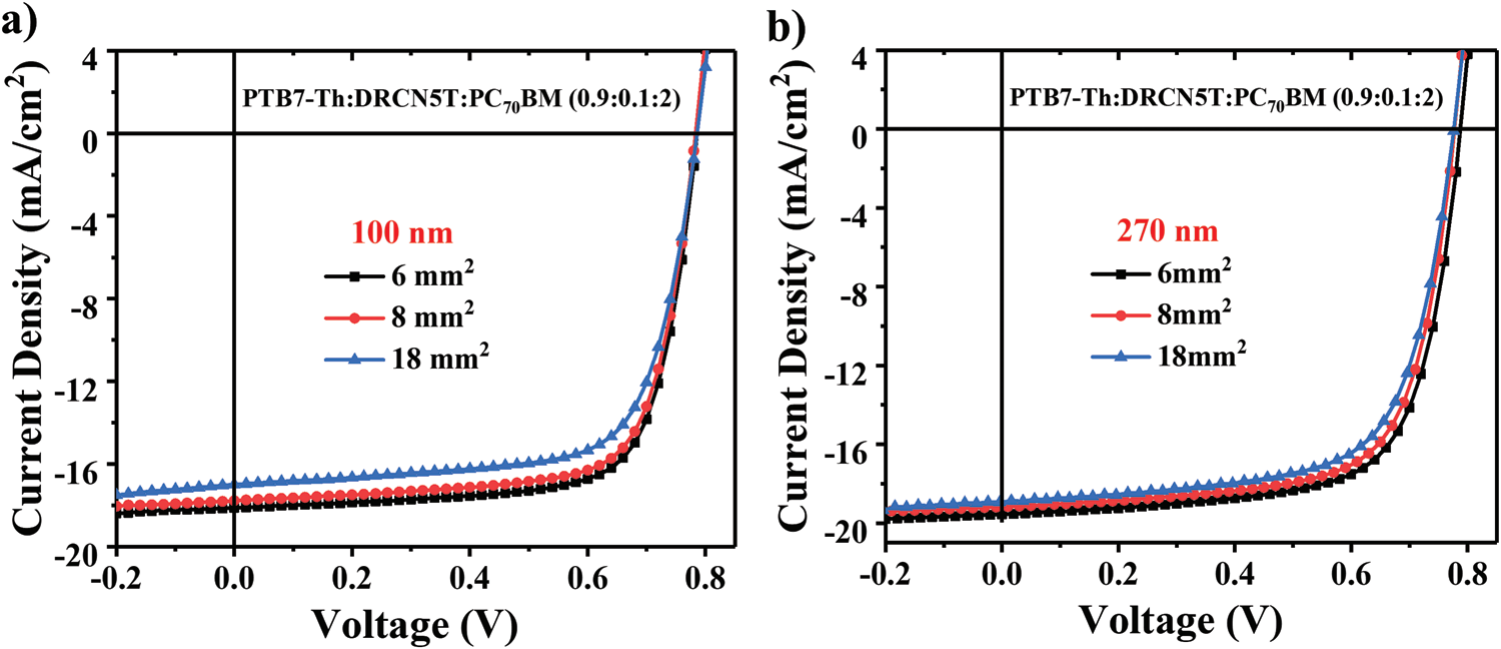
*J*–*V* curves of PTB7‐Th:DRCN5T:PC_70_BM devices with active layer thickness of a) 100 nm and b) 270 nm at different efficient area.

To further demonstrate the possibility that the incorporation of DRCN5T could induce the crystallization of PTB7‐Th for fabrication of devices with thick blend film, the binary and ternary OSCs incorporating 10 wt% DRCN5T with the blend film thickness changing from 180 to 350 nm were prepared, and the corresponding results are summarized in Table S1 and Figure S2c,d in the Supporting Information. 270 nm was observed to be the optimal ternary blend film thickness, exhibiting the highest PCE value at 11.1% (Table 1). The high device performance was mainly owing to the significant increase of *J*_sc_ value from 18.63 to 20.10 mA cm^−2^, although the FF decreased from 73.5% to 69.8%. Increasing the blend film thickness to 350 nm reduced FF to 60.7% and *J*_sc_ to 18.75 mA cm^−2^, achieving an acceptable PCE value of 8.94%. In contrast, in the PTB7‐Th:PC_70_BM binary system, when the blend film thickness was 250 nm, the *J*_sc_ increased to 17.16 mA cm^−2^ while the FF dropped to 57.4%, resulting in a low PCE value of 7.78%. Further increasing to 350 nm eventually leads to the sharply reduced *J*_sc_, FF, and PCE values of 9.25 mA cm^−2^, 49.6%, 3.69%, respectively. In comparison to the binary systems, the ternary system containing 10 wt% DRCN5T exhibited an improvement of PCE by 29.1% at blend film thickness of 100 nm and 42.7% at blend film thickness of 250 nm, respectively. These results are similar to those reported by other research groups,^31^, ^40^, ^54^ suggesting that the incorporation of crystalline molecule could facilitate the fabrication of PTB7‐Th:PC_70_BM‐based devices with thick active layer suitable for large‐area printing. The optimal devices with different area of 6, 8, and 18 mm^2^ were prepared to explore the impact of efficient area on the corresponding performance, the detailed results are summarized in **Figure** **3** and **Table** **2**. Both of the devices containing 10 wt% DRCN5T with blend film thickness of 100 and 270 nm exhibited an excellent PCE value over 10% as the efficient area was increased from 6 to 18 mm^2^.

**Table 2:** Photovoltaic performance of optimal thin and thick ternary blend films with different active layer areas

PTB7‐Th:DRCN5T: PC_70_BM (0.9:0.1:2)	Area [mm^2^]	*V*_oc_ [V]	*J*_sc_ [mA cm^−2^]	FF [%]	PCE_max_ [%]
100 nm	6	0.785	18.12	72.8	10.4
	8	0.785	17.98	72.3	10.2
	18	0.783	17.89	71.9	10.1
	6	0.779	19.53	69.6	10.6
270 nm	8	0.777	19.19	69.2	10.3
	18	0.776	18.93	68.8	10.1

The external quantum efficiency (EQE) spectra of these OSCs were exhibited in Figure S2b in the Supporting Information. The integrating current density provided by EQE curves are illustrated in Table 1 and Table S1 in the Supporting Information, all these values are conformed to the *J*_sc_ obtained from the *J*–*V* curves with errors less than 3%. For the binary blend systems, the EQE values were improved between 500 and 650 nm as blend film thickness was increased from 100 to 250 nm, which corresponds with the absorption spectra in Figure S1b in the Supporting Information. Unfortunately, the EQE value obviously reduced between 650 and 750 nm, leading to the poor *J*_sc_. For the ternary blend systems with blend film thickness increasing from 100 to 270 nm, the absorption enhancement from 500 to 800 nm plays a great role in the improvement of EQE value, which is contributed by DRCN5T and PTB7‐Th. These results might be originating from the change of vertical gradient distribution of different components in the blend film,^21^ which will be explained in the later section.

**Figure 4:**
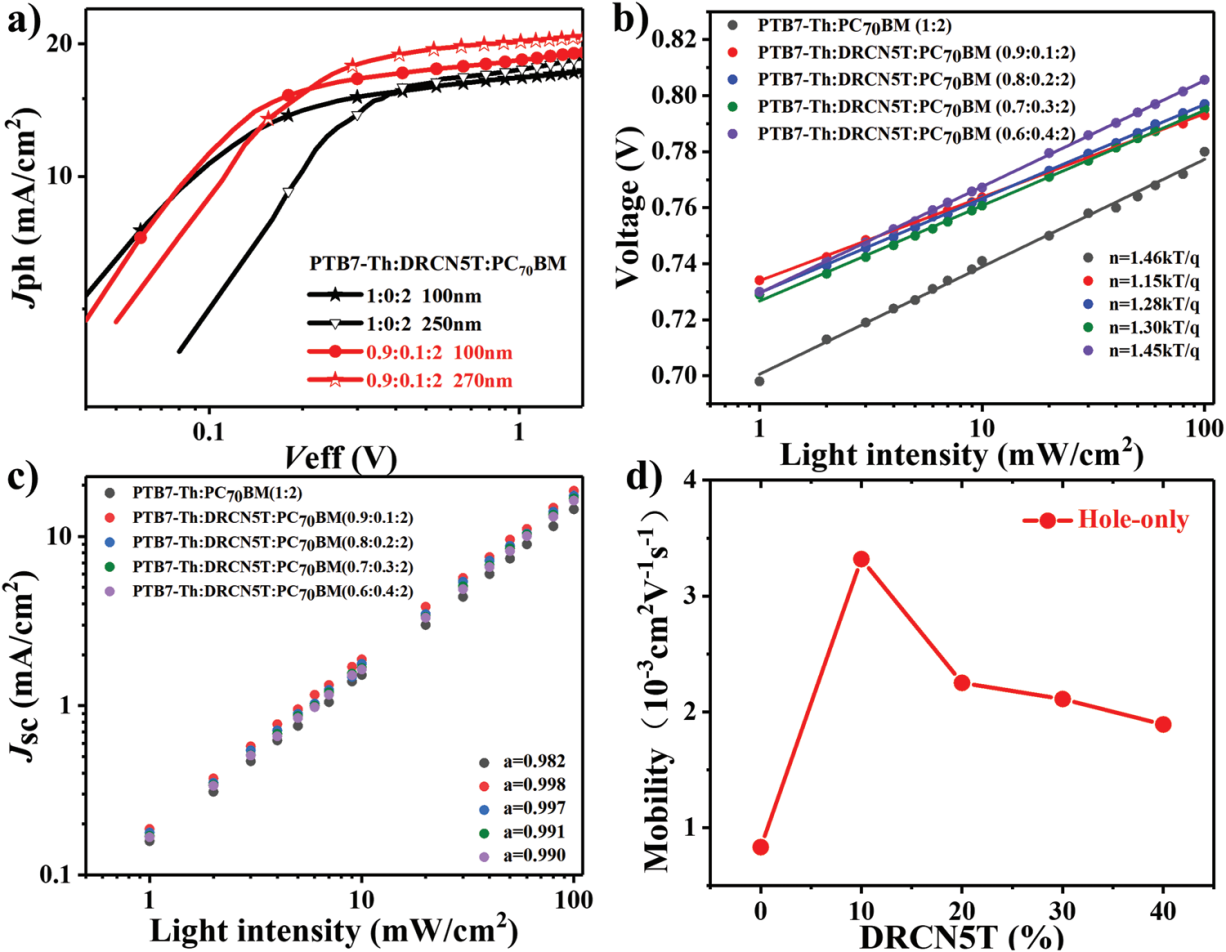
a) Photocurrent density versus effective voltage (*J*_ph_–*V*_eff_) curves of devices with thin and thick active layers, b) dependence of *V*_oc_ and c) *J*_sc_ on light intensity for the ternary OSCs with different DRCN5T ratios, d) hole mobility versus DRCN5T ratio in devices with thin active layers.

Hoping to further explore the function of DRCN5T ratio on the performance of ternary OSCs, density of photocurrent versus voltage (*J*_ph_–*V*_eff_) curves of all OSCs are carried out and displayed in **Figure** **4**a and Figure S3 in the Supporting Information. Here, the detail definitions of *J*_ph_ and *V*_eff_ are discussed in Electronic Supporting Information. For PTB7‐Th:PC_70_BM binary devices, the *J*_sat_ values were 17.82 mA cm^−2^ for 100 nm thick active layer and 18.92 mA cm^−2^ for 250 nm thick active layer. For ternary devices, the *J*_sat_ were 19.42 mA cm^−2^ (10 wt% DRCN5T, 100 nm), 20.93 mA cm^−2^ (10 wt% DRCN5T, 270 nm), 18.85 mA cm^−2^ (20 wt% DRCN5T, 100 nm), 18.37 mA cm^−2^ (30 wt% DRCN5T, 100 nm), and 18.26 mA cm^−2^ (40 wt% DRCN5T, 100 nm), respectively. It was obvious that the ternary systems with both of thin and thick active layers show prominent enhancement of *J*_sat_ as compared with PTB7‐Th:PC_70_BM system. Moreover, the *J*_sat_ of ternary system (10 wt% DRCN5T) exhibited prominent improvement when the blend film thickness was increased to 270 nm and is 17.5% higher than that of the PTB7‐Th:PC_70_BM binary system with thin active layer. The result indicated that the usage of thick active layer is one of the efficient methods to improve the *J*_sc_ by enhancing light harvesting. The *J*_ph_^d^/*J*_sat_ and *J*_ph_^c^/*J*_sat_ expound the differences of these devices.^34^, ^55^ Here, the *J*_ph_^d^ and *J*_ph_^c^ refer to the current density at short circuit and maximum power output conditions, respectively. The exciton dissociation efficiency (η_d_) and charge collection efficiency (η_c_) can be assessed by *J*_ph_^d^/*J*_sat_ and *J*_ph_^c^/*J*_sat_. The detailed *J*_sat_, *J*_ph_^d^, *J*_ph_^c^, η_d_, and η_c_ values of these OSCs are summarized in Tables S2 and S3 in the Supporting Information. The η_d_ for the DRCN5T ternary devices are more highlighted than those for binary OSCs. The η_d_ and η_c_ of ternary OSCs increased first and then decreased along with the increment of DRCN5T ratio, indicating that the phase separation extent of ternary blend films can be changed by incorporating DRCN5T content.^43^, ^56^ Furthermore, the ternary OSCs (10 wt% DRCN5T, 270 nm) still maintained a high η_d_ and η_c_ of 96% and 83%, respectively, whereas the binary system (250 nm) decreased to 90% and 78%. The result indicated that the incorporation of DRCN5T not only improved the light absorption but also promoted the exciton dissociation. The optimized ternary OSCs exhibited the maximal η_d_ of 96% and η_c_ of 85%, which was consistent with the largest FF of 73.6%.

To elucidate charge transport dynamics in blend films, the *J*–*V* curves of these OSCs are measured under different illumination intensity from 1 to 100 mW cm^−2^, as shown in Figure 4 and Figure S4 in the Supporting Information. The relationship between *J*_sc_ and light intensity could be expressed by the power‐law formula of *J*_sc_ ∝ *P*_light_ *^α^*, where α is the exponential factor.^57^ Generally, the α value is approximately equal to 1, indicating the fewest bimolecular recombination of active layer. The α value was 0.982 for PTB7‐Th:PC_70_BM system, which was lower than that of the ternary systems with different DRCN5T content. For the optimized ternary OSCs, the highest α value of 0.998 was calculated, which was very close to 1, suggesting the weakest bimolecular recombination.^57^ Moreover, the *V*_oc_ dependence on light intensity could be assessed by *V*_oc_ ∝ *n* (*KT*/*q*)ln (*P*_light_)_,_ *n* value is generally ranging from 1 to 2. The *n* value close to 1 indicates the insignificant trap‐assisted recombination in blend film. The *n* value reaching to 2 suggests the serious trap‐assisted recombination causing by impurities or defects in blend films.^6^, ^34^ The *n* values were 1.15 (10 wt% DRCN5T), 1.28 (20 wt% DRCN5T), 1.30 (30 wt% DRCN5T), and 1.45 (40 wt% DRCN5T) for the ternary OSCs, which is smaller than the *n* value of 1.46 for the binary PTB7‐Th:PC_70_BM system. The *n* value was close to 1 of the optimized ternary OSCs, suggesting the negligible trap‐assisted recombination in this system.^56^ Both of these two recombinations can be effectually suppressed for more charge transport and then collected in the ternary OSCs after the incorporation of 10 wt% DRCN5T into PTB7‐Th:PC_70_BM system, which could be further verified by the larger η_c_ of 85% and FF of 73.6% for the optimal ternary OSCs.

As is known, it has already been reported that balanced charge transport and high charge mobility strongly affect the performance of OSCs, especially for thick film devices. To further study the performance of OSCs with both thin and thick blend films, the charge mobilities were measured by utilizing space charge–limited current method. The curves of single‐charge devices under dark mode are revealed in Figure 4d and Figure S3a–c in the Supporting Information. The calculated hole mobility (*µ*_h_) and electron mobility(*µ*_e_) were shown in Table S4 in the Supporting Information. First, for the binary and ternary OSCs with active layer thickness of about 100 nm, the *µ*_h_ and *µ*_e_ of PTB7‐Th:PC_70_BM binary devices were 8.31 × 10^−4^ and 6.18 × 10^−4^ cm^2^ V^−1^ s^−1^, respectively. When DRCN5T was added into PTB7‐Th:PC_70_BM, the *µ*_h_ and *µ*_e_ values of all the ternary devices are greater than those in binary devices. Furthermore, both of the *µ*_h_ and *µ*_e_ values reached to the maximum up to 3.32 × 10^−3^ and 2.85 × 10^−3^ cm^2^ V^−1^ s^−1^ when the DRCN5T content was 10 wt%. The value of *µ*_h_/*µ*_e_ was calculated to be 1.16, indicating the more balanced charge transport, which is beneficial to the higher FF and PCE. As compared with binary blend systems, the more excellent charge transfer of ternary blend systems should be due to the optimized morphology upon the incorporation of DRCN5T. Furthermore, the mobility of PTB7‐Th:PC_70_BM and PTB7‐Th:DRCN5T:PCBM (0.9:0.1:2)‐based devices with the blend film thickness of 250 and 270 nm was determined. It was observed that the *µ*_h_ value of binary blend systems apparently reduces to 6.51 × 10^−4^ cm^2^ V^−1^ s^−1^ as the active layer thickness increases to 250 nm. However, in the ternary blend systems, a decrease in hole mobility from 3.32 × 10^−3^ to 2.20 × 10^−3^ cm^2^ V^−1^ s^−1^ is noticed. The variation in FF of the OSCs mentioned above is consistent with the mobility change of holes and electrons. The higher mobility of charges and more balanced *µ*_h_/*µ*_e_ value facilitate to improve charge extraction and restrain charge recombination, which could well explain the fact that binary OSCs with thicker films show a lower PCE than the thinner ones while the ternary OSCs (10 wt% DRCN5T) with thicker films exhibiting a higher PCE.

**Figure 5:**
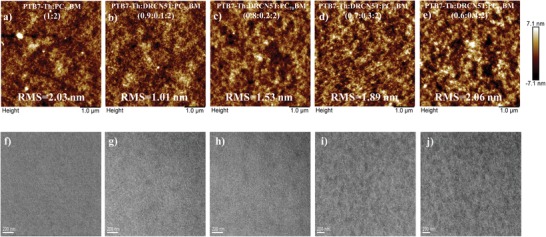
a–e) AFM and f–j) TEM images of blend films with different DRCN5T ratio.

Based on the above analysis, the significant improvement of photovoltaic performance of PTB7‐Th:PC_70_BM system upon incorporation of DRCN5T should be related to the optimized morphology of blend films including the beneficial phase separation in horizontal and vertical directions, as well as the crystallization of polymers. Thus, atomic force microcopy (AFM) and transmission electron microscopy (TEM) techniques were performed to investigate the surface and internal morphology of the blend films,^50^ and the corresponding images are displayed in **Figure** **5** and Figure S5 in the Supporting Information. From the AFM images, the ternary blend films (100 nm) showed a more uniform morphology and smoother surface than the PTB7‐Th:PC_70_BM film (100 nm), exhibiting root mean square (RMS) surface roughness of 1.01 nm (10 wt% DRCN5T), 1.53 nm (20 wt% DRCN5T), 1.89 nm (30 wt% DRCN5T), and 2.06 nm (40 wt% DRCN5T), respectively. As the blend films thickness increases to 180 and 250 nm or 270 nm, the binary blend films exhibited a rougher surface (RMS = 2.83 nm for 180 nm, RMS = 3.44 nm for 250 nm, respectively). In contrast, the ternary blend films showed of a smooth surface (RMS = 1.21 nm for 180 nm, RMS = 1.36 nm for 270 nm, respectively), which is slightly larger than the above 100 nm thick ternary blend film (10 wt% DRCN5T). It is suggested that the difference of RMS value in binary and ternary blend films might be relevant to the dissimilar surface behavior causing by the change in vertical composition and miscibility between different components.^22^, ^44^

In the TEM images, it was observed that the obvious nanofibrous structure appears after incorporation of DRCN5T into PTB7‐Th:PC_70_BM, providing more channels for charge generation and transport, which is beneficial to improving the photovoltaic performance.^58^ The appearance of nanofibers induced by DRCN5T also demonstrated the more ordered structure of PTB7‐Th, contributing to the shift of absorption from 709.9 to 724.6 nm, which was in accordance with UV spectra. With the increase of DRCN5T content, the density of the nanofibers increased, accompanied by the appearance of notable phase separation at DRCN5T ratio above 30 wt%. Further increasing the thickness of blend films from 100 to 270 nm leads to the gradual morphology evolution in PTB7‐Th:PC_70_BM binary blend films, showing discernable phase separation structure, which is consistent with the sharp depression in *J*_sc_ and FF. In ternary active layers (10 wt% DRCN5T), the morphology of film at 180 and 270 nm seems to be the same, exhibiting discernable fibrous structure. It is suggested that the DRCN5T could act as nucleating agent to induce the crystallization of PTB7‐Th and stabilize the morphology as the thickness of the films increases.

**Figure 6:**
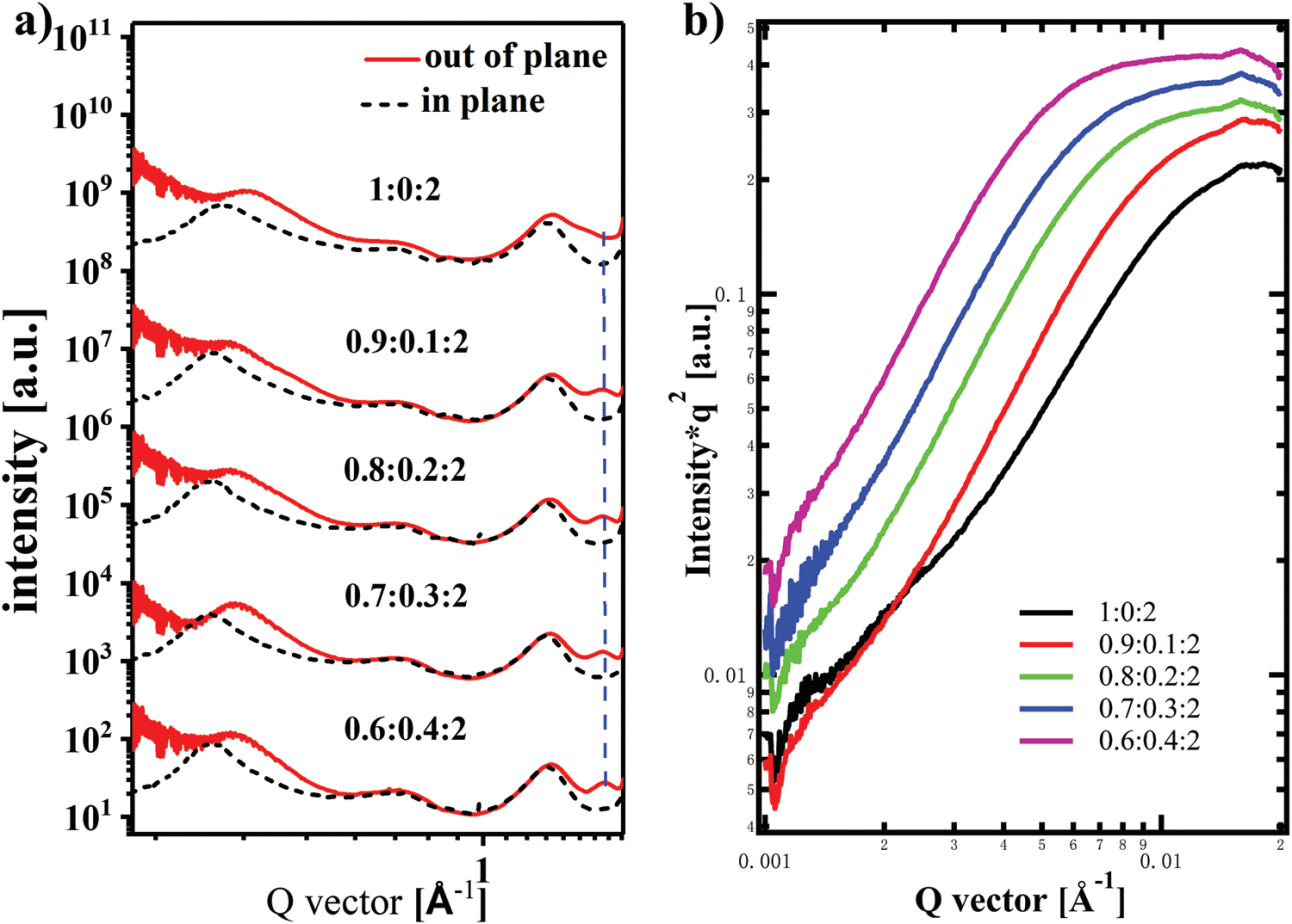
a) Out‐of‐plane and in‐plane curves and b) R‐SoXS profiles for PTB7‐Th:DRCN5T:PC_70_BM ternary blend films.

Then, the crystalline structure of the films with various DRCN5T contents was investigated by GIWAXS,^21^ and the corresponding 2D patterns and 1D cuts in the in‐plane and out‐of‐plane directions are shown in Figure S6 in the Supporting Information and **Figure** **6**a, and the corresponding parameters are displayed in Table S5 in the Supporting Information. Generally, the (100) lamellar peak of PTB7‐Th at 0.34 Å^−1^ in the out‐of‐plane curve is noticed for PTB7‐Th:PC_70_BM binary blend film, in addition to the obvious peak at 0.31 Å^−1^ in the in‐plane curve. By the incorporation of 10 and 20 wt% DRCN5T into the binary system, the (100) peaks slightly shift to lower *q* values, and the lamellar distance between alkyl chains became larger. Moreover, the coherence length of ternary blend system significantly increased in contrast to the binary PTB7‐Th:PC_70_BM blend, indicating that DRCN5T could promote the crystallization of PTB7‐Th, in accordance with the UV and TEM result. Similarly, the (010) peak at 1.61 Å^−1^ is less obvious in the PTB7‐Th:PC_70_BM blend, and the d‐spacing is determined to be 3.90 Å. Upon the addition of DRCN5T, the d‐spacing of (010) peak significantly reduced, suggesting of stronger π–π stacking. The corresponding coherence length of the ternary blend film is larger than that in binary active layer, indicating that the addition of DRCN5T could induce the crystallization of PTB7‐Th and formation of more face‐on crystals, which is consistent with the appearance of interconnected fibrous structure and obviously improved hole mobility. Additionally, the aggregation size of PC_70_BM slightly increased upon the incorporation of DRCN5T, which is probably due to the bad miscibility between DRCN5T and PC_70_BM molecules.

Resonant soft X‐ray scattering (R‐SoXS) was also implemented on investigating the domain size of these different active layer films, and the Lorentz‐corrected R‐SoXS scattering profiles of various DRCN5T ratios active layer films are shown in Figure 6b. The domain size (Table S5, Supporting Information) of 17.0 nm (*q* = 0.018 Å^−1^) is observed for PTB7‐Th:PC_70_BM blend film, which was increased to 19.8 nm as the DRCN5T weight ratio increases from 10 to 40 wt%. Moreover, the scattering intensity gradually increased with the incorporation of small molecules, showing that the enhanced crystallinity correlates with the higher domain purity. The larger domain size demonstrates that the incorporation of DRCN5T could drive the PC_70_BM molecules into larger aggregates.

**Figure 7:**
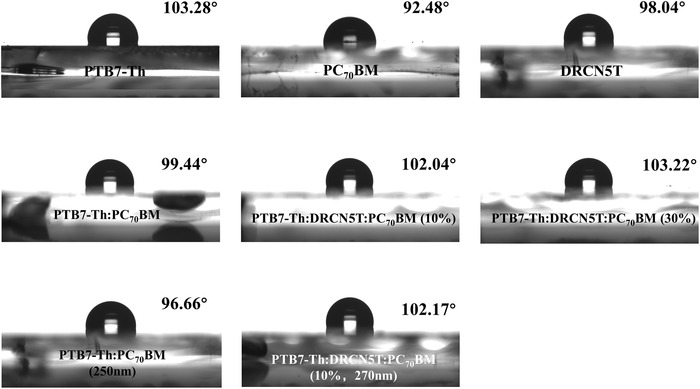
WCA images of pristine a) PTB7‐Th, b) PC_70_BM, c) DRCN5T films, in addition to the thin d) binary and ternary blend films with e) 10% and f) 30% DRCN5T, the thick g) binary and h) ternary blend films with 10% DRCN5T.

As discussed previously, the incorporation of DRCN5T to improve the photovoltaic performance of PTB7‐Th:PC_70_BM‐based devices originates from two aspects, including facilitating the crystallization of PTB7‐Th to form interconnected fibrous structure and optimizing donor and acceptor distribution in vertical direction. Therefore, a water contact angle (WCA) test of pristine and blending films of PTB7‐Th, PC_70_BM, DRCN5T, PTB7‐Th:PC_70_BM (100 and 250 nm), PTB7‐Th:DRCN5T:PC_70_BM (0.9:0.1:2) (100 and 270 nm), PTB7‐Th:DRCN5T:PC_70_BM (0.7:0.3:2) (100 nm) was performed to explore the effect of small molecules on the surface behaviors of active layer, and the corresponding results are appeared in **Figure** **7**. Due to the different surface energies of PTB7‐Th and PC_70_BM, the WCAs test can visually reflect the distribution of donor or acceptor in the blend film. The WCAs are determined to be 103.28°, 92.48°, 98.04° of PTB7‐Th, PC_70_BM, and DRCN5T, respectively. It is indicated that PTB7‐Th donor has a lower surface energy, and it is more intense hydrophobic than PC_70_BM and DRCN5T. As the blending films are concerned, only one component should be distributed on the film surface if the WCA value is close to the single‐component pure film. In Figure 7, it is found that the WCA value is 99.44° of PTB7‐Th:PC_70_BM, which is close to the pure film of PTB7‐Th (103.28°), indicating that more PTB7‐Th gather on the blend film superficially, in addition to the small amount of PC_70_BM molecules. For the thin ternary blend films, the WCA values increased gradually after the incorporation of DRCN5T. The WCA values are 102.04° of ternary films containing 10 wt% DRCN5T and 103.22° of ternary films containing 30 wt% DRCN5T, which are very close to the pure PTB7‐Th film of 103.28°. As the thick films are concerned, the WCA values exhibit distinct changes, showing a WCA value of 96.66° for binary blend film and 102.17° for ternary blend film (10 wt% DRCN5T), respectively. In contrast to the thin PTB7‐Th:PC_70_BM film (100 nm), the thick binary blend film (250 nm) shows a much smaller WCA, attributing to the enrichment of more PC_70_BM molecules on the surface of films. As the ternary PTB7‐Th:DRCN5T:PC_70_BM (0.9:0.1:2) film is concerned, the WCA value of the thick film (270 nm) is very close to that of the thin film (100 nm). These results indicate that more PC_70_BM enriched on the surface of the thick binary blend film while more PTB7‐Th enriched on the surface of thin binary film and ternary films, which well correlates with the EQE result in Figure 2a. Based on these data, it is included that the incorporation of DRCN5T into PTB7‐Th:PC_70_BM could alter the vertical distribution of PTB7‐Th and PC_70_BM, which is vital in the charge collection as well as the eventual photovoltaic performance. Moreover, the vertical distribution of donor or acceptor influencing by the DRCN5T should be due to the difference in miscibility between DRCN5T and donor/acceptor, which has never been reported previously.^28^, ^44^ The miscibility between the different components is recognized as the key factor in determining the vertical and horizontal phase separation, and eventually the performance of solar cells. Anyway, how miscible between DRCN5T and PC_70_BM is actually unknown.^51^

The interaction parameter χ from the melting point depression method based on the DSC equipment could be calculated by Equation (1)

(1) $$\frac{1}{T_{m}}\;\;-\;\;\frac{1}{T_{m}^{0}}\;\;=\;\;-\frac{R}{\Delta H_{f}}\frac{\nu_{2}}{\nu_{1}}\left[\frac{\ln\phi_{2}}{m_{2}}\;\;+\;\;\left(\frac{1}{m_{2}}\;\;-\;\;\frac{1}{m_{1}}\right)\;\;\;\times \;\;\;(1\;\;-\;\;\phi_{2})\;\;+\;\;\chi {\left(1\;\;-\;\;\phi_{2}\right)}^{2}\right]$$

where subscript 1 is identified with the weaker crystalline material and 2 with the stronger crystalline material, respectively. *T*_m_ and *T*_m_^0^ are the melting points of mixture and pure crystalline material. *R* is the ideal gas constant. Δ*H*_f_ is the heat fusion of crystalline material. The ν is the molar volumes. The *m* is the degree of polymerization. Φ is the volume fraction. For the PTB7‐Th:DRCN5T blends, the subscript 1 represents the weakly crystalline polymer PTB7‐Th and 2 identifies the highly crystalline DRCN5T, *m*_1_ is very large compared to 1 and *m*_2_ is 1. Then, the above equation reduces to Equation (2)

(2) $$\frac{1}{T_{m}}\;\;-\;\;\frac{1}{T_{m}^{0}}\;\;=\;\;-\frac{R}{\Delta H_{f}}\;\;\frac{\nu_{2}}{\nu_{1}}\;\;\left[\ln\;\;\phi_{2}\;\;+\;\;(1\;\;-\;\;\phi_{2})\;\;+\;\;\chi {\left(1\;\;-\;\;\phi_{2}\right)}^{2}\right]$$

For the DRCN5T:PC_70_BM blends, the subscript 1 identifies with the less crystalline molecule PCBM and 2 with the highly crystalline molecule DRCN5T, both of *m*_1_ and *m*_2_ are 1, the above equation is thus reduced to Equation (3)

(3) $$\frac{1}{T_{m}}\;\;-\;\;\frac{1}{T_{m}^{0}}\;\;=\;\;-\frac{R}{\Delta H_{f}}\frac{\nu_{2}}{\nu_{1}}\;\;\left[\ln\;\;\phi_{2}\;\;+\;\;\chi {\left(1\;\;-\;\;\phi_{2}\right)}^{2}\right]$$

By neglecting the effects of entropy and Φ_2_, the χ can have the following form

(4) $$\chi =\frac{B\nu_{1}}{RT}$$

where *B* is the interaction energy density characteristic of the organic material pair.

Substituting Equation (4) into the above equations, the χ can be extracted by the slope of the linear fit straight line.

The DSC heating and cooling curves of DRCN5T:PTB7‐Th and DRCN5T:PC_70_BM blends are exhibited in Figure S7 in the Supporting Information, the corresponding melting temperatures are listed in **Table** **3**. In the PTB7‐Th:DRCN5T blends, the χ value is calculated to be about −0.80. In contrast, the χ value in DRCN5T:PC_70_BM blends is determined to be about 2.94, as listed in **Table** **4**. The negativeness of χ for the PTB7‐Th:DRCN5T blend indicates that they can form a thermodynamically stable and compatible mixture at temperatures above the melting point. Additionally, the DRCN5T and PC_70_BM are totally immiscible.

**Table 3:** The melting temperatures of DRCN5T with the increase of PTB7‐Th or PC_70_BM ratio

DRCN5T:PTB7‐Th	$T_{m}^{0}$_DRCN5T_ [°C]	DRCN5T:PC_70_BM	$T_{m}^{0}$_DRCN5T_ [°C]
10:0^a)^	219.6	10:0^a)^	219.6
9:1	219.1	9:1	207.2
7:3	217.9	7:3	207.6
5:5	215.7	5:5	204.7
3:7	N/A	3:7	200.6
0:10	N/A	0:10	N/A

^a)^ Δ*H*_DRCN5T_ = −44.89 J g^−1^.

**Table 4:** Parameters for the calculation of Flory–Higgins interaction parameters by the melting‐point depression method

Blends	$T_{m}^{0}$ [°C]	*v*_1_ [cm^3^ mol^−1^]	*v*_2_ [cm^3^ mol^−1^]	Δ*H*_f_ [J mol^−1^]	χ (*T*_m_)
DRCN5T:PTB7‐Th	219.6	772.2	1000.7	45 873	−0.80
DRCN5T:PC_70_BM	219.6	381.8	1000.7	45 873	2.94

Therefore, the incorporation of the DRCN5T serves several roles to optimize the morphology for eventual increase of photovoltaic performance. One is the nucleating agent, the crystalline DRCN5T could induce the crystallization of PTB7‐Th polymers to form the interconnected fibrous network, facilitating the transport of charges. Second, based on the surface energy and the interaction parameters, DCNR5T molecules should be well blended in the PTB7‐Th phase, residing in the interface between PTB7‐Th and PC_70_BM. The bad miscibility between DRCN5T and PC_70_BM eventually drives PC_70_BM to separate from PTB7‐Th phase, leading to the enrichment of PTB7‐Th on the active layer surface. In other words, the DRCN5T molecules could optimize the vertical phase separation, leading to enrichment of PTB7‐Th at the anode and PC_70_BM at the cathode.

## 3 Conclusions

In summary, high‐efficient ternary OSCs have been designed and prepared. As compared with binary system, the ternary device with 10 wt% DRCN5T displayed a highest PCE of 11.1% whose blend film thickness is 270 nm. Encouragingly, the high‐performance ternary devices with both of thin and thick films showing active layer area of 18 mm^2^ could still achieve PCE above 10%. The superior performance is mainly due to improved charge collection and separation, attributing to the formation of obvious interpenetrating network morphology. Another important reason is that DRCN5T could optimize the vertical distribution of PTB7‐Th and PC_70_BM because of different miscibility with donor or acceptor. The miscibility could be quantitatively described by Flory–Huggins interaction parameter, which were calculated to be −0.80 and 2.94 in DRCN5T:PTB7‐Th and DRCN5T:PC_70_BM blends, demonstrating that DRCN5T and PTB7‐Th are miscible and DRCN5T and PC_70_BM are immiscible. The poor miscibility between DRCN5T and PC_70_BM eventually led to the enrichment of PTB7‐Th on the active layer surface. The finding in this work provides novel guideline to select appropriate third additive based on the miscibility between different components.

## 4 Experimental Section

*Materials*: DRCN5T, PTB7‐Th, and PC_70_BM were acquired from Solarmer Materials Inc. Chlorobenzene and other solvent were purchased from Sigma Co. Ag, Al, and MoO_3_ were purchased from Zhong Nuo Advanced Materials Co. In order to ensure the accuracy of experimental results, all the materials employed in the experiment were used from the same batch.

*Device Fabrication*: Both binary and ternary active layers were fabricated with inverted device structures to evaluate the performance of PTB7‐Th:PC_70_BM host binary system and PTB7‐Th:DRCN5T:PC_70_BM ternary system with various DRCN5T contents. The concentration of the solution is 20 mg mL^−1^ and the donor and acceptor weight ratio is maintained at 1:2, in addition to the volume ratio of solvent (CB) and additive (1, 4‐butanediol, BT) keeping at 97:3. The solutions were stirred at 70 °C overnight. The ITO glass substrates (≤15 Ω sq^−1^) were cleaned successively via acetone, ultrasonication in liquid detergent, deionized water, and isopropanol for 30 min of each step. ZnO precursor solution was prepared by dissolving 0.5 g of zinc acetate dihydrate (Zn(CH_3_COO)_2_ ⋅ 2H_2_O, 99%) in 5 mL of 2‐methoxyethanol (CH_3_OCH_2_CH_2_OH, 98%) and 0.138 mL of ethanolamine (NH_2_CH_2_CH_2_OH, 98%), and stirring overnight at room temperature.^50^ The cleaned ITO glass substrates were dried by nitrogen flow and then treated by UV‐Ozone for 18 min. Then, the ZnO precursor solution was spinned at 4000 rpm for 1 min and annealed at 200 °C for 60 min in air atmosphere to afford 30 nm thick layer, followed by transferring the substrates into the N_2_ glove box for spinning the active layers. The thickness of active layer was kept at around 100 nm. For the thicker film, the concentration of solution was increased to 35 mg mL^−1^ and the spin rate was ranging from 600 to 1800 rpm to afford films with different thickness. After thermal evaporation of 7 nm MoO_3_ and 90 nm Ag under a pressure of 2 × 10^−4^ Pa, the devices were then used for further characterization. For the common devices, the active area was kept at 4 mm^2^. For the devices with larger area, the ITO width was increased from 2 to 9 mm with the same shadow mask.

*Device and Film Characterizations*: The solar simulator (SS‐F5‐AAA, Enli technology Co., Ltd.) with an AM 1.5 G (100 mW cm^−2^) spectra was used to perform the *J*–*V* measurements. The intensity of spectra was calibrated by the certified standard silicon solar cell (SRC‐2020, Enli technology Co., Ltd.). The EQE data were obtained by using solar cell spectral‐response measurement (QE‐R, Enli technology Co., Ltd.). The UV‐vis absorption spectra were obtained by Hitachi UV‐3010 spectrophotometer. The morphology of blend films was measured by AFM (Nanoscope III A scanning probe microscope) and TEM (JSM‐7100F).

## Conflict of Interest

The authors declare no conflict of interest.

## Supporting information

Supplementary
