# Supplementary material for: Miscibility Tuning for Optimizing Phase Separation and Vertical Distribution toward Highly Efficient Organic Solar Cells
Source: Adv Sci (Weinh). 2019 May 22;6(15):1900565. doi: 10.1002/advs.201900565 (PMC6685468; doi:10.1002/advs.201900565)
Supplement: Supplementary file 1 — Supplementary [file ADVS-6-1900565-s001.pdf]

Copyright WILEY-VCH Verlag GmbH & Co. KGaA, 69469 Weinheim, Germany, 2019.

ADVANCED SCIENCE
Open Access

Supporting Information

for *Adv. Sci.,* DOI: 10.1002/advs.201900565

Miscibility Tuning for Optimizing Phase Separation and Vertical Distribution toward Highly Efficient Organic Solar Cells

Lifu Zhang, Nan Yi, Weihua Zhou, Zoukangning Yu, Feng Liu,* and Yiwang Chen**

Supporting Information

Miscibility Tuning for Optimizing Phase Separation and Vertical Distribution Toward Highly Efficient Organic Solar Cells

Lifu Zhang, Nan Yi, Weihua Zhou Zoukangning Yu, Feng Liu*, Yiwang Chen**

L. Zhang, Prof. Y. Chen

College of Chemistry, Nanchang University

Institute of Polymers and Energy Chemistry (IPEC), Nanchang University, 999 Xuefu Avenue, Nanchang 330031, China

E-mail: ywchen@ncu.edu.cn

Prof. W. Zhou, N. Yi, Z. Yu

School of Material Science and Engineering, Nanchang University

Institute of Polymers and Energy Chemistry (IPEC), Nanchang University, 999 Xuefu Avenue, Nanchang 330031, China

E-mail: zhouweihua@ncu.edu.cn

Prof. F. Liu

School of Chemistry and Chemical Engineering, Shanghai Jiaotong University, 800 Dongchuan, Shanghai 200240, China

E-mail: Fengliu82@sjtu.edu.cn

1. Measurements and Characterizations

J_{ph} – V_{eff} Measurement: Here, J_{ph} is defined as $J_{ph} = J_L - J_D$, J_L and J_D are the current density under illumination and in dark. V_{eff} is defined as $V_{eff} = V_0 - V$, V_0 is the voltage at which J_{ph} is equal to zero; V is the applied bias voltage.[1]

V_{oc} Dependence on Light Intensity: The V_{oc} dependence on light intensity could be assessed by natural logarithmic formula of $V_{oc} \propto$ n (KT/q)ln (P_{light}), where n is a slope value, K is Boltzmann's constant and T is absolute temperature, q is elementary charge.[2]

Hole and electron mobility (SCLC) measurement: The electron-only and hole-only devices were built with the structures of ITO/ZnO/active layers/Al and ITO/PEDOT:PSS/active layers/MoO_3/Ag, respectively. which can be defined as:

$$J = 9\varepsilon_r\varepsilon_0\mu V^2/8L^3$$

where J is the current density under dark mode, ε_r is the dielectric constant relative of the transport medium (assumed to be 3), ε_0 is the permittivity of free space, μ referred to μ_h or μ_e, V is the voltage in device which is defined as $V = V_{appl}$-V_r-V_{bi}, V_{appl} is the applied voltage (-5V~5V), V_r is the voltage drop across the electrodes, and V_{bi} is the built-in voltage. L is the thickness of the active layer (100 nm, 250 nm, 270 nm).

Grazing-incidence wide-angle X-ray scattering (GIWAXS): GIWAXS measurements were performed at the Advanced Light Source (ALS), Berkeley, USA. Samples were prepared on Si substrates by using identical blend solutions as those used in devices. The incident angle was 0.14°, which maximized the scattering intensity from the samples. The scattering X-rays were detected by using a Dectris Pilatus 2M photon counting detector.[3]

Resonant Soft X-Ray Scattering (R-SoXS): R-SoXS measurements were performed

at the ALS. Samples for R-SoXS measurements were prepared on a PEDOT:PSS covered ITO substrate under the same conditions as those used for device fabrication, and then transferred by floating in water to a 1.5 mm × 1.5 mm, 100 nm thick Si_3N4 membrane supported by a 5 mm × 5 mm, 200 μm thick Si frame (Norcada Inc.). 2D scattering patterns were collected on an in-vacuum CCD camera (Princeton Instrument PI-MTE). The sample detector distance was calibrated from diffraction peaks of a triblock copolymer poly (isoprene-b-styrene-b-2-vinyl pyridine), which has a known spacing of 391 Å. The beam size at the sample was about 100 μm × 200 μm. We measured the resonant soft X-ray scattering (R-SoXS) using 284.8 eV photon energy. The characteristic mode length scale ξ (the distance between two donor domains or two acceptor domains) was estimated by the equation of $\xi = 2\pi/q$ mode, and the mode domain size is the half of ξ.[4]

DSC measurement and the calculation of interaction parameters (χ): Differential scanning calorimetry (DSC) was measured by TA DSC Q2000 differential scanning calorimeter, with the samples being heated to 300 °C and then cooled to 40 °C at a heating/cooling rate of 10 °C/min.[5]

2. Supplementary Information

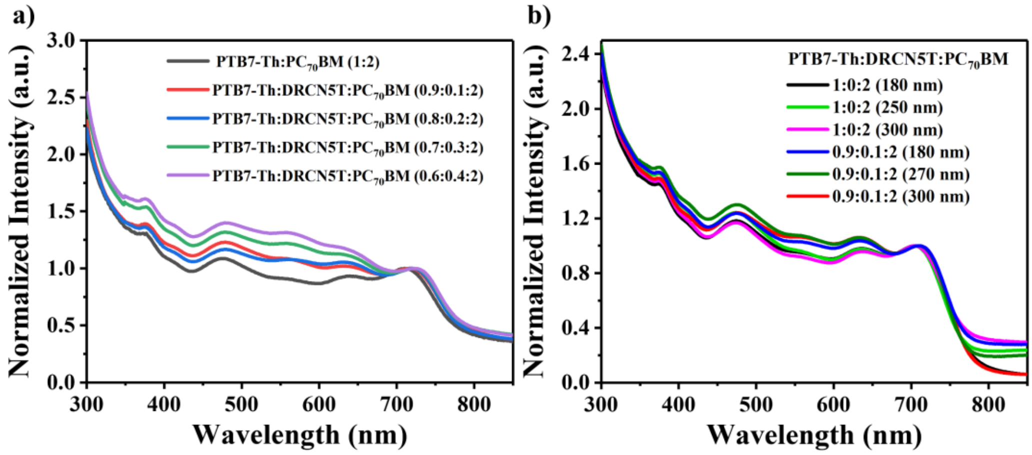

Figure S1. Absorption spectra of binary and ternary blend films under different conditions, (a) the thickness of the active layer is about 100 nm, (b) the thickness of the active layer ranging from 180 nm to 300 nm.

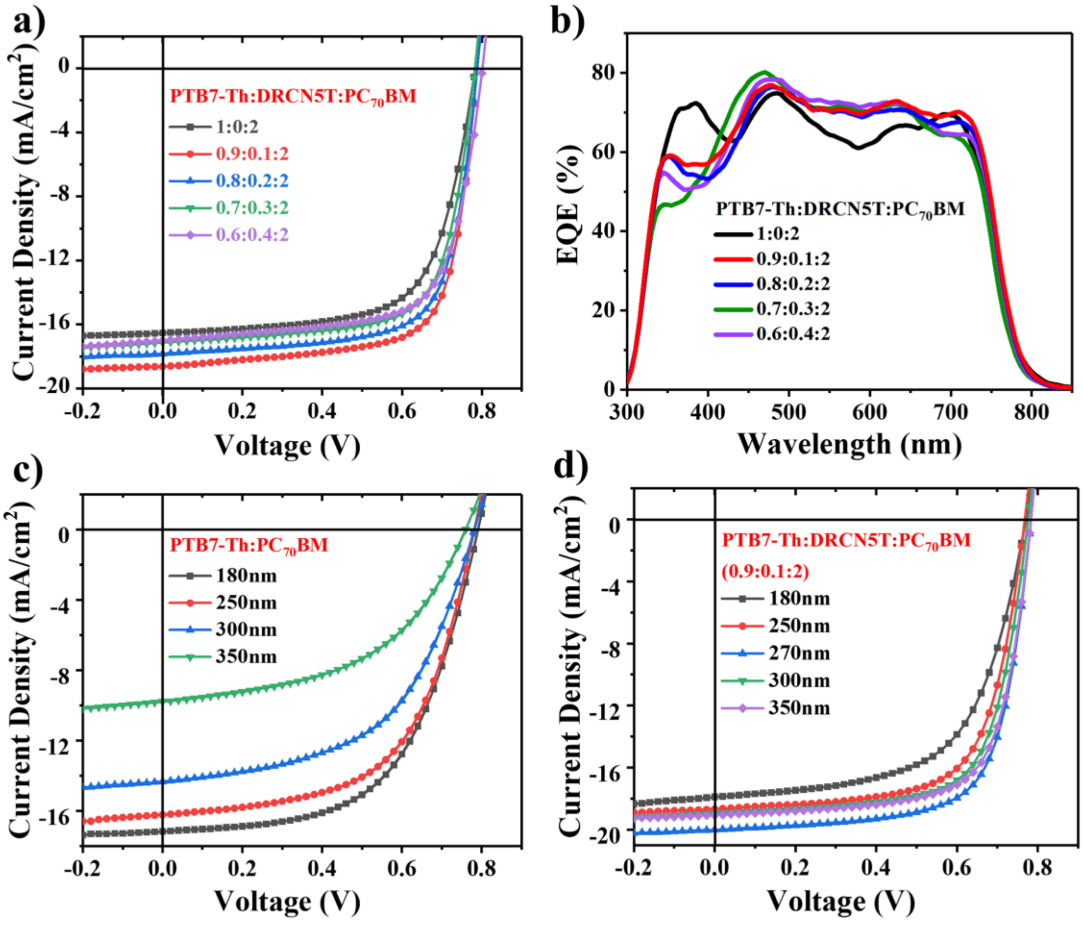

Figure S2. (a) *J-V* and (b) EQE curves of OSCs based on PTB7-Th:DRCN5T:$PC_{70}BM$ ternary devices versus DRCN5T content. The *J-V* curves of (c) binary PTB7-Th:$PC_{70}BM$ and (d) ternary PTB7-Th:DRCN5T:$PC_{70}BM$ devices with differently thick active layer.

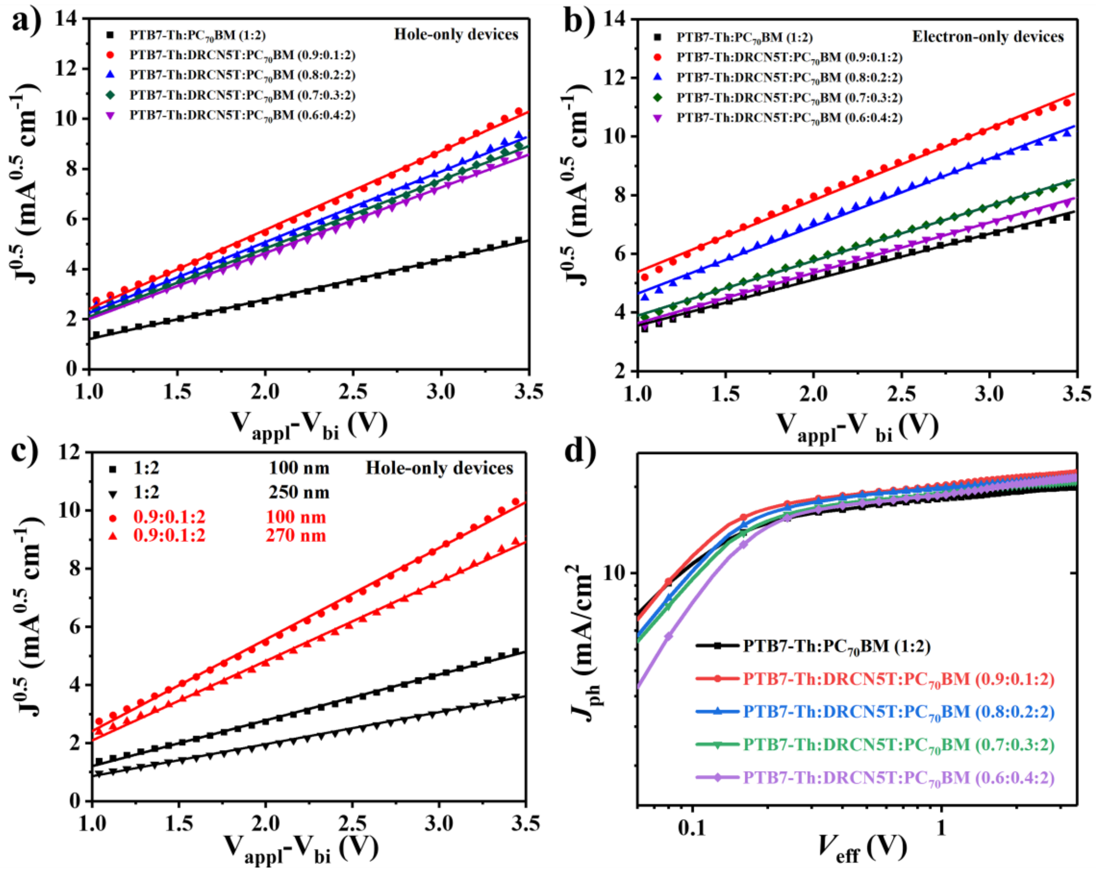

Figure S3. (a) Hole-only and (b) electron-only devices with different DRCN5T content. (c) Hole-only devices of binary and ternary blend films with different thickness. (d) The photocurrent density versus effective voltage ($J_{ph} - V_{eff}$) curves of devices with binary and ternary blend films.

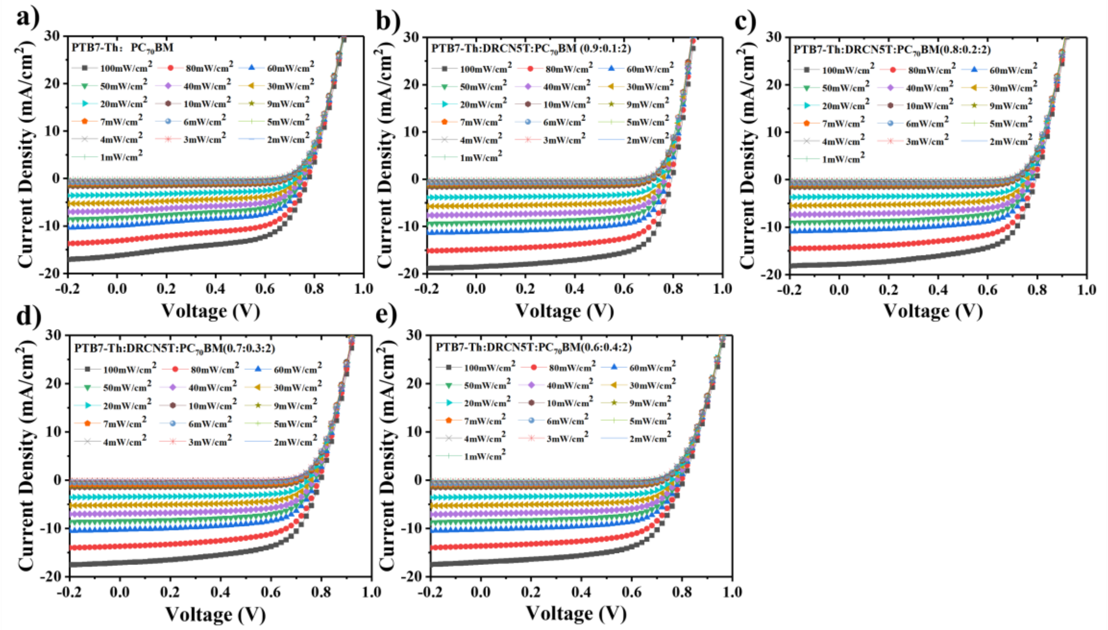

Figure S4. *J*-*V* curves of binary and ternary OSCs under different light intensity.

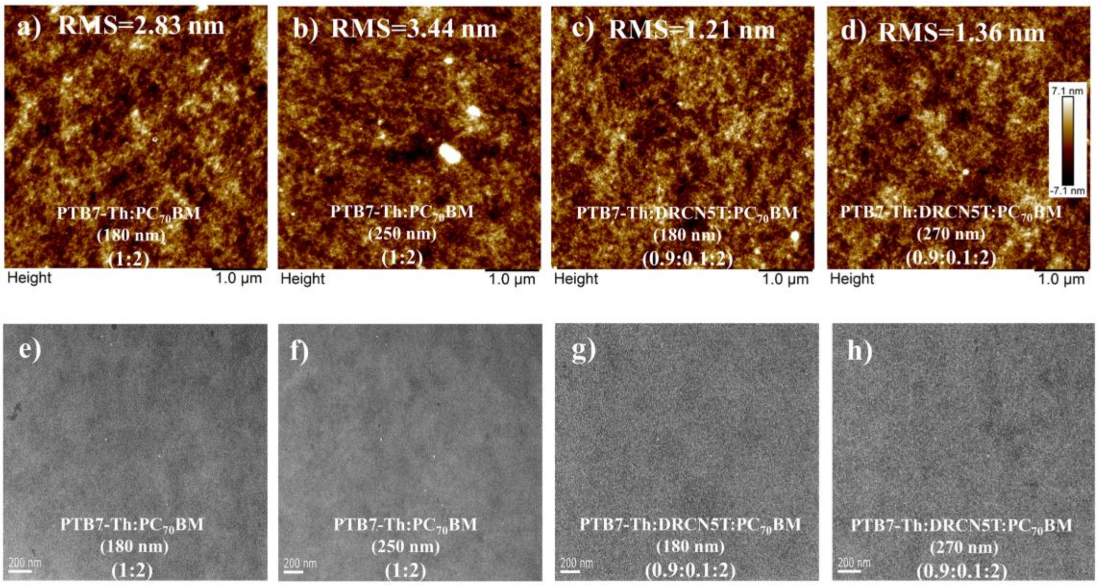

Figure S5. (a-d) AFM and TEM (e-h) images of blend films with different thickness.

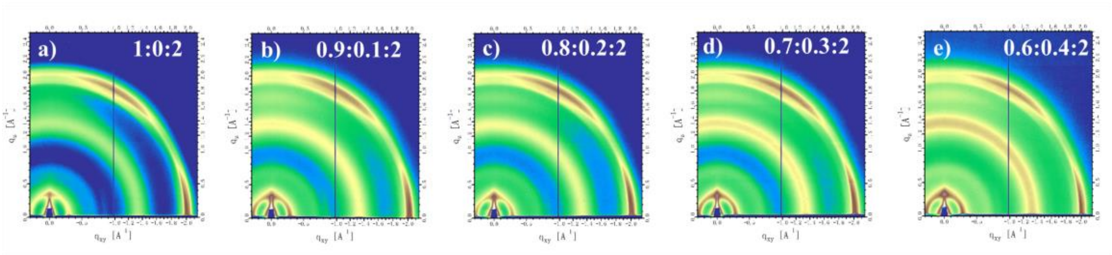

Figure S6. 2D grazing incidence wide angle X-ray scattering (GIWAXS) patterns of blend films with different DRCN5T ratio.

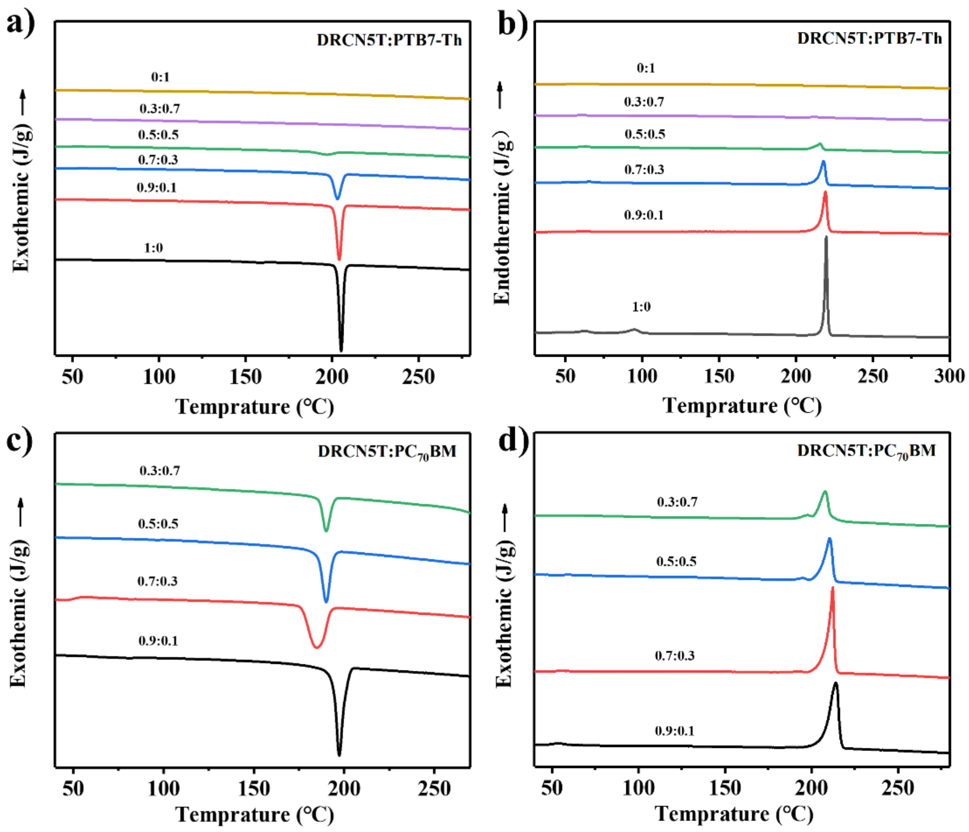

Figure S7. The DSC heating and cooling curves of (a, b) DRCN5T:PTB7-Th blends, and (c, d) DRCN5T:$PC_{70}BM$ blends.

Table S1. Photovoltaic parameters of binary and optimal ternary OSCs with active layer thickness increasing from 180 nm to 350 nm.

**PTB7-Th:DRCN5T: $PC_{70}BM$**	**Thickness [nm]**	**V_{oc} [V]**	**J_{sc} [mA cm^{-2}]**	**J_{calcd} [mA cm^{-2}]**	**FF [%]**	**PCE_{ave} [%]**	**PCE_{max} [%]**
**1:0:2**	180	0.79±0.002	16.21±0.03	15.9	57.8±0.3	7.3±0.2	7.4
	250	0.78±0.002	17.16±0.04	16.4	57.4±0.3	7.8±0.2	7.9
	300	0.78±0.001	14.48±0.01	13.9	53.2±0.1	5.9±0.3	6.2
	350	0.76±0.003	9.79±0.02	9.25	49.6±0.3	3.7±0.2	3.8
**0.9:0.1:2**	180	0.78±0.001	18.00±0.07	17.53	70.0±0.4	9.8±0.1	10.0
	250	0.78±0.002	18.86±0.07	17.95	68.6±0.5	10.1±0.1	10.2
	270	0.78±0.002	20.10±0.07	19.05	69.8±0.2	10.7±0.4	11.1
	300	0.77±0.006	19.05±0.07	18.20	67.1±0.3	9.7±0.1	9.8
	350	0.77±0.004	19.24±0.05	18.75	60.7±0.2	8.9±0.3	9.1

Table S2. Photoelectric parameters of OSCs with different DRCN5T ratio.

PTB7-Th:DRCN5T:$PC_{70}BM$	(1:0:2)	(0.9:0.1:2)	(0.8:0.2:2)	(0.7:0.3:2)	(0.6:0.4:2)
J_{sat}	17.82	19.42	18.85	18.37	18.26
J_{ph}^{d}	16.53	18.625	17.84	17.13	16.99
J_{ph}^{c}	14.52	16.46	15.90	15.25	15.11
J_{ph}^{d} / J_{sat}	0.93	0.96	0.95	0.93	0.93
J_{ph}^{c} / J_{sat}	0.82	0.85	0.84	0.83	0.83

Table S3. Photoelectric parameters of OSCs with binary and ternary active layers of different thickness.

	PTB7-Th:PC$_{70}$BM (1:0:2)		PTB7-Th:DRCN5T:PC$_{70}$BM (0.9:0.1:2)	
	**100 nm**	**250 nm**	**100 nm**	**270 nm**
J_{sat}	17.82	18.99	19.42	20.93
J_{ph}^{d}	16.53	17.16	18.63	20.10
J_{ph}^{c}	14.52	14.82	16.46	17.42
J_{ph}^{d} / J_{sat}	0.93	0.90	0.96	0.96
J_{ph}^{c} / J_{sat}	0.82	0.78	0.85	0.83

Table S4. Hole and electron mobility for PTB7-Th:DRCN5T:$PC_{70}BM$ thin and thick thickness blend films with various DRCN5T contents.

**PTB7-Th:DRCN5T:PC_{70}BM**	μ_h **(cm^2 V^{-1} s^{-1})**	μ_e **(cm^2 V^{-1} s^{-1})**	μ_h **/**μ_e
**1:0:2 (100 nm)**	8.31×10^{-4}	6.18×10^{-4}	1.34
**1:0:2 (250 nm)**	6.51×10^{-4}	/	/
**0.9:0.1:2 (100 nm）**	3.32×10^{-3}	2.85×10^{-3}	1.16
**0.9:0.1:2 (270 nm）**	2.20×10^{-3}	/	/
**0.8:0.2:2 (100 nm)**	2.25×10^{-3}	1.78×10^{-3}	1.26
**0.7:0.3:2 (100 nm)**	2.11×10^{-3}	1.71×10^{-3}	1.23
**0.6:0.4:2 (100 nm)**	1.89×10^{-3}	1.49×10^{-3}	1.27

Table S5. Data of GIWAXS and R-SoXS characterization for of PTB7-Th:DRCN5T:$PC_{70}BM$ active layer films with different DRCN5T ratio.

	**(100)**			**(010)**			**$PC_{70}BM$ aggregation**		**R-SoXS**	
PTB7-Th: DRCN5T: $PC_{70}BM$	peak location ($Å^{-1}$)	*d*-spacing (Å)	coherence length (Å)	peak location ($Å^{-1}$)	*d*-spacing (Å)	coherence length (Å)	peak location ($Å^{-1}$)	coherence length (Å)	peak location ($Å^{-1}$)	domain size (nm)
1:0:2	0.34	18.38	93.78	1.61	3.90	38.60	1.37	23.92	0.018	17.0
0.9:0.1:2	0.32	19.87	142.80	1.70	3.69	39.11	1.36	25.18	0.016	19.8
0.8:0.2:2	0.32	19.62	123.20	1.71	3.67	42.06	1.35	26.20	0.016	19.8
0.7:0.3:2	0.32	19.36	273.18	1.71	3.67	39.96	1.35	25.72	0.016	19.8
0.6:0.4:2	0.32	19.36	241.66	1.73	3.62	47.46	1.35	26.33	0.016	19.8

References

[1] Y. Chen, T. Liu, H. Hu, T. Ma, J. Y. L. Lai, J. Zhang, H. Ade and H. Yan, *Adv. Energy Mater.* **2018**, 8, 1801203.

[2] Y. Xie, F. Yang, Y. Li, M. A. Uddin, P. Bi, B. Fan, Y. Cai, X. Hao, H. Y. Woo, W. Li, F. Liu and Y. Sun, *Adv. Mater.* **2018**, 30, 1803045.

[3] Z. Zhou, S. Xu, J. Song, Y. Jin, Q. Yue, Y. Qian, F. Liu, F. Zhang and X. Zhu, *Nat. Energy* **2018**, 3, 952.

[4] L. Zhang, X. Xu, B. Lin, H. Zhao, T. Li, J. Xin, Z. Bi, G. Qiu, S. Guo, K. Zhou, X. Zhan and W. Ma, *Adv. Mater.* **2018**, 30, 1805041.

[5] Q. Ai, W. Zhou, L. Zhang, L. Huang, J. Yin, Z. Yu, S. Liu, W. Ma, J. Zeng and Y. Chen, *J. Mater. Chem. C* **2017**, *5*, 10801.
